# Characteristics and trends of spontaneous reporting of therapeutic ineffectiveness in South Korea from 2000 to 2016

**DOI:** 10.1371/journal.pone.0212905

**Published:** 2019-02-28

**Authors:** Hye-Jun Kim, Han Eol Jeong, Ji-Hwan Bae, Yeon-Hee Baek, Ju-Young Shin

**Affiliations:** School of Pharmacy, Sungkyunkwan University, Suwon, Gyeonggi-do, South Korea; University of Kansas Medical Center, UNITED STATES

## Abstract

Therapeutic ineffectiveness involves drug-related therapeutic failure, inefficacy or resistance and has not been sufficiently studied. Objective of our study was to evaluate reporting trends in therapeutic ineffectiveness by year and describe factors affecting therapeutic ineffectiveness using the Korea Adverse Event Reporting System. Proportion of therapeutic ineffectiveness reports was based on total submitted reports between 2000 and 2016. Utilizing 2016 alone, we compared the characteristics of therapeutic ineffectiveness with age group and gender matching by random extraction. We conducted a logistic regression analysis to estimate reporting odds ratios (ROR) and its 95% confidence intervals (CI) for reports by type of reporters, e.g., doctors, pharmacists, or consumers. We presented most frequent reports by the anatomical main groups and therapeutic subgroups according to the Anatomical Therapeutic Chemical (ATC) classification system. For the 17-years, the proportion of therapeutic ineffectiveness adverse drug reactions reporting ranged from 0.0% to 3.7% between 2000 and 2016. Of 228,939 reports, 2,797 (1.2%) were submitted in 2016. Consumers accounted for 6.92% of reports and doctors accounted for 45.49%, in which, consumers were more likely to report therapeutic ineffectiveness than doctors (adjusted ROR 3.98; 95% CI, 2.92 to 5.41). According to the ATC classification system, “nervous system” was the most frequently reported anatomical group (18.7%) and “parathyroid hormones and analogues” was reported most frequently in the pharmacological subgroup (23.7%). Teriparatide, a drug used to treat osteoporosis, had the most reports (11.0%). Therapeutic ineffectiveness reports may be used as a scientific tool for the reevaluation of respective drugs in order to confirm of its therapeutic effects.

## Introduction

“Therapeutic ineffectiveness” is medicinal ineffectiveness that includes terms such as “drug ineffective”, “inefficacy”, “effect, lack of” or “ineffectiveness” [1]. Unlike controlled clinical trials where subjects are a well-defined and selected population, real-world settings are based on real-life populations, resulting in drug performance that is often unpredictable [2]. Therapeutic ineffectiveness is a common drug-related problem that often involves therapeutic failure, inefficacy or resistance. 16% of hospital admissions occurred due to drug-related problems, of which, more than half (55%) were from therapeutic failure [3].

Although therapeutic ineffectiveness includes both unintended and potentially harmful responses [2], these reports also potentially contribute to identifying pharmaceutical defects of drugs [1] from a different point of view. Therapeutic ineffectiveness issues may also be related to quality in manufacturing processes. These pharmaceutical defects, including failure of quality control in line with good manufacturing practices or improperly stored and/or transported drugs, may lead to therapeutic ineffectiveness.

Nevertheless, studies associated with therapeutic ineffectiveness are lacking worldwide. A few review studies have covered the definition of therapeutic ineffectiveness, but no studies have dealt with the proportion of reports regarding therapeutic ineffectiveness. In addition, there are no previous studies concerning which drugs are reported as being therapeutic ineffectiveness, who is primarily reporting the therapeutic ineffectiveness (doctor, pharmacist, or consumer), or what age group and gender chiefly report therapeutic ineffectiveness.

Thus, our objective for this study was to assess the therapeutic ineffectiveness reports in South Korea by determining trends regarding time, gender, age, and the Anatomical Therapeutic Chemical (ATC) classification system in the therapeutic ineffectiveness reports, as well as identifying factors affecting therapeutic ineffectiveness using the Korea Adverse Event Reporting System (KAERS) database.

## Materials and methods

### Data sources

The spontaneous adverse drug reactions (ADRs) reporting system in South Korea was introduced in 1988 and the online reporting system was initiated in 2000; the system is regulated by the Ministry of Food and Drug Safety (MFDS). In 2012, the pharmacovigilance (PV) activities were transferred to the Korea Institute of Drug Safety & Risk Management (KIDS), which developed the KIDS-KAERS database [4]. Our study used the KAERS database, established by the KIDS in South Korea (Data number: 1707A0044). KIDS forbids the transfer, rent, or sale of the database to any third party other than the researcher, who obtained the approval for the provided database (Official website of KIDS: http://open.drugsafe.or.kr/; Contact information of data access committee: +82-2-2172-6700). We accessed the data used in our study in the above mentioned manner, which we expect future researchers to do so in the same manner, and did not receive special privileges from KIDS.

A total of 1,089,163 reports have been accrued in the KAERS database between December 1988 and June 2016. We acquired all reports of therapeutic ineffectiveness between 2000 and 2016 to determine any temporal trends in the reports (Data number: 1801A0002). Additionally, we acquired the KAERS database for 2016 (Data number: 1707A0044).

The total eligible therapeutic ineffectiveness-related and non-related reports were randomly selected from 228,939 reports (735,370 drug-adverse event pairs) in the 2016 KAERS database. In addition, we analyzed the trends in the reporting of therapeutic ineffectiveness, which were analyzed based on the year the reports were recorded in the KAERS database from 2000–2016. Reports missing gender or age group data were excluded from our study.

### Definition of therapeutic ineffectiveness

We included reports that were eligible for therapeutic ineffectiveness. Therapeutic ineffectiveness is a term of the World Health Organization Adverse Reaction Terminology (WHO-ART) used by the Uppsala Monitoring Centre in the WHO’s Programme for International Drug Monitoring. Therapeutic ineffectiveness, in our study, included the following seven terms: medicine ineffective, drug ineffective, inefficacy, ineffectiveness, effect, lack of, anesthesia insufficient, or medicine ineffective unexpected [5].

### 17-year longitudinal reporting trends of therapeutic ineffectiveness

The reporting trends for therapeutic ineffectiveness over the 17 years from 2000 to 2016 were graphed. The horizontal axis shows the year and vertical axis shows all reported ADRs, proportions, and proportion of therapeutic ineffectiveness.

### Covariates definition

When comparing characteristics of therapeutic ineffectiveness and non-therapeutic ineffectiveness, we utilized categories of the KAERS database to control potential confounders for therapeutic ineffectiveness. Age group, gender, report source by person, report source by affiliation and serious adverse events (AE) were used for reports to calculate frequency and proportion.

Age groups were classified into six age subgroups: Neonates, aged <28 days; Infants, aged 28 days to 2 years; Children, aged 2 to 12 years; Adolescents, aged 12 to 19 years; Adults, aged 19 to 65 years; Elderly, aged ≥65 years. Report source by person was classified into the following: doctor, pharmacist, nurse, consumer, other, and medical expert (one who practices one branch of medicine). Report source by affiliation was categorized into: regional PV center (RPVC), pharmaceutical company, medical institution, pharmacy, health center, consumer, and other. For serious AEs, seriousness was defined as including any of the following six terms: death, life-threatening, hospitalization, persistent or significant disability/impairment, congenital defects/anomalies or other medically important conditions [6, 7].

### Characteristics of therapeutic ineffectiveness

To describe the characteristics of therapeutic ineffectiveness reports versus those not recorded as therapeutic ineffectiveness, we chose comparable groups by applying age group and gender matched AE reports. We included all AE reports recorded as therapeutic ineffectiveness and others not recorded as therapeutic ineffectiveness. We used 1 to 1 exact matching for age group and gender by random extraction.

### Utilization of the ATC classification system

We utilized the ATC classification system from the WHO Collaborating Centre for Drug Statistics Methodology (https://www.whocc.no/atc_ddd_index/). The ATC classification system consists of the following five levels: anatomical main group, therapeutic subgroup, pharmacological subgroup, chemical subgroup, and chemical substance. To determine the proportion of ATC classification system subgroups involved in therapeutic ineffectiveness, we utilized the anatomical main groups and therapeutic subgroups in ATC classification system. We also determined the most frequently reported drug in 2016.

### Statistical analysis

We analyzed frequencies and percentages for categorical variables. The chi-square test was used to compare categorical variables and a *p-value* < 0.05 was considered to be statistically significant. To calculate reporting odds ratios (RORs) and their 95% confidence intervals (CI) for being reported as a therapeutic ineffectiveness, logistic regression analysis was performed by controlling for potential confounders: age group, gender, report source by person, report source by affiliation, and serious adverse event. For age- and gender-matched data, conditional logistic regression analysis was done. For both logistic and conditional logistic regression analysis, an automated/statistical criteria, also known as stepwise, was utilized. To evaluate to goodness of fit for the final model, the c-statistic was used for calculation of the predictive accuracy, which was found to be 0.859. All statistical procedures were performed using SAS 9.4 software (SAS Institute Inc., Cary, NC, USA). The study protocol was approved by the Institutional Review Board (IRB) of Sungkyunkwan University (IRB No. SKKU 2018-03-019) and obtaining informed consent from the study population was waived by the board.

## Results and discussion

**Fig 1:**
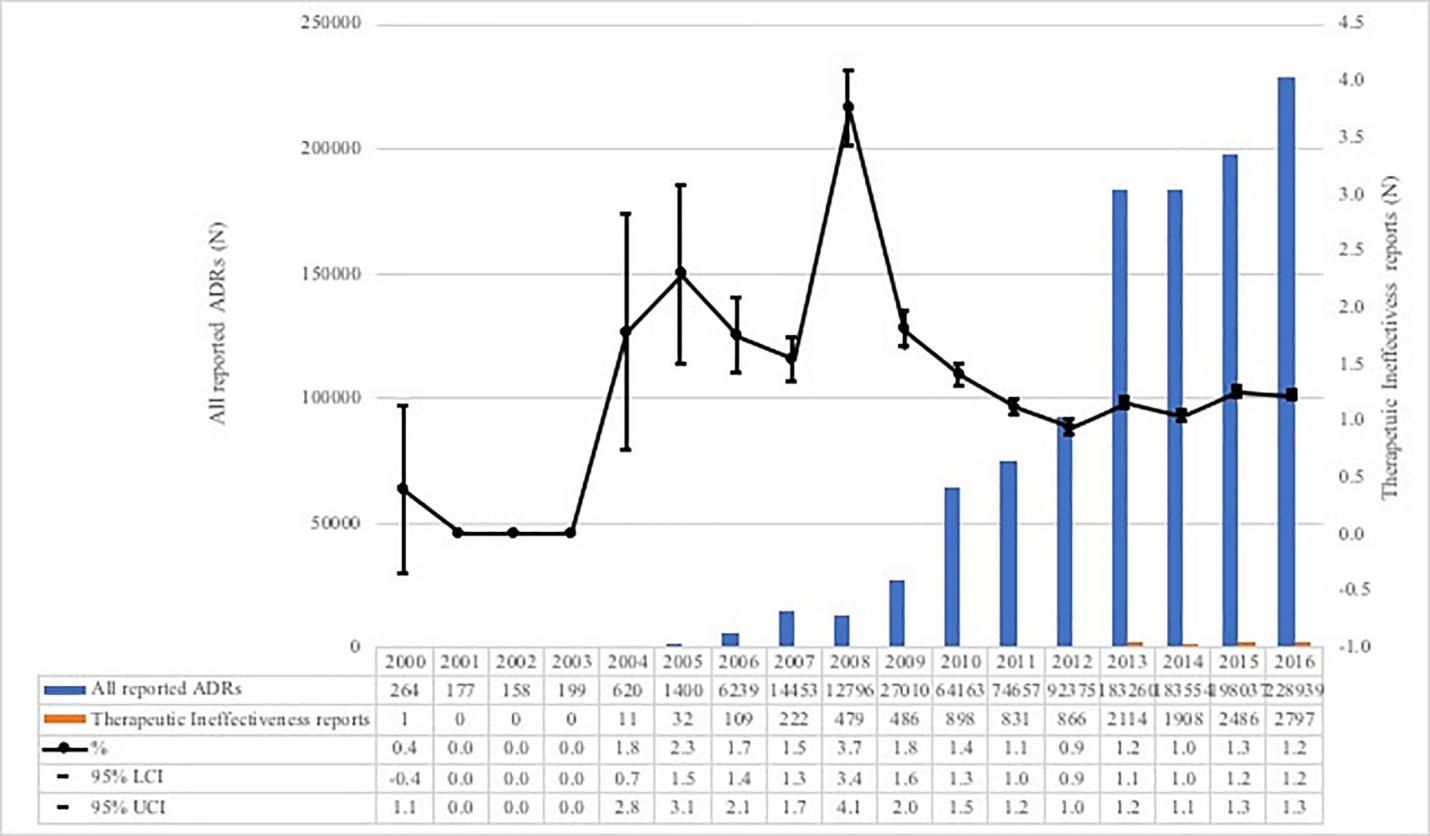
Trends and proportion of therapeutic ineffectiveness reports compared with all reported ADRs from the KAERS database between January 2000 and December 2016.

**Fig 2:**
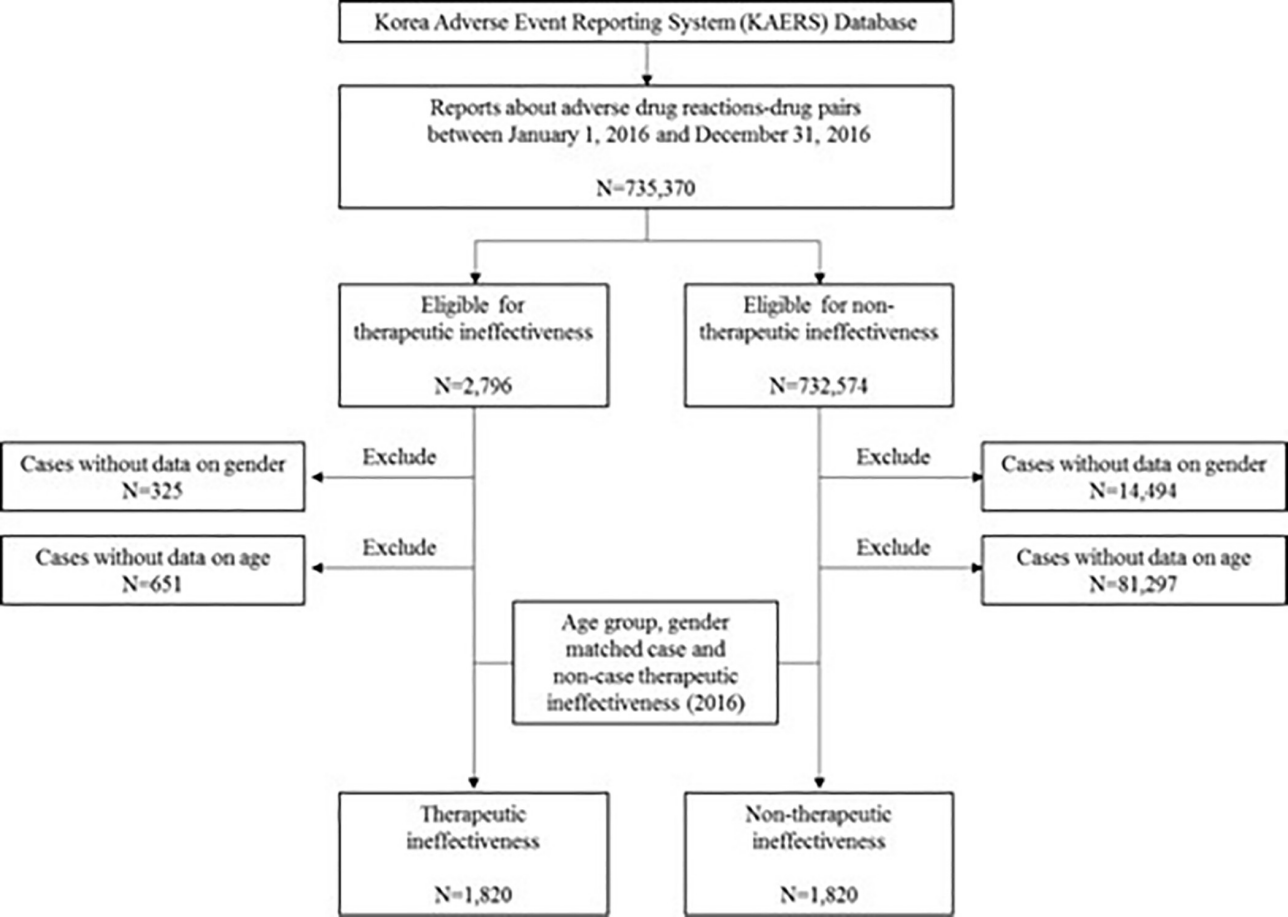
Therapeutic and non-therapeutic ineffectiveness searching process in the KAERS database from 2016.

For the entire study period of 17-years (2000–2016), proportion of therapeutic ineffectiveness ranged from 0.0% to 3.7% (Fig 1). As for therapeutic ineffectiveness characterization in 2016 only, we identified a total of 2,797 (1.2%) therapeutic ineffectiveness reports. To compare the characteristics of reports, we categorized 1,820 age- and gender-matched reports of therapeutic ineffectiveness and 1,820 non- therapeutic ineffectiveness related reports (Fig 2).

Table 1 describes the characteristics of therapeutic and non-therapeutic ineffectiveness reports in 2016. After age group and gender matching, therapeutic ineffectiveness reports accounted for 61.15% for adults and 33.46% for the elderly. Additionally, 50.16% and 49.84% of the reports were reported by females and males, respectively. Regarding the report source by person, 45.49% of the reports were presented by doctors, 6.92% by consumers, and 409 reports were missing this variable (22.47%). As for report source by affiliation, 44.23% were reported by pharmaceutical companies and 0.93% by consumers. Overall, 18.96% of reports were identified as reports with a serious AE.

**Table 1:** Characteristics of therapeutic and non-therapeutic ineffectiveness applied to age group- and gender-match reports received from the KAERS database in 2016.

	Before age group, gender matching									After age group, gender matching^d^																		
	TI Reports				Non-TI Reports				*p-value*	TI Reports									Non-TI Reports									*p-value*
	N = 2,796 (%)				N = 732,574 (%)					N = 1,820 (%)				95% CI					N = 1,820 (%)				95% CI					
**Age group**^**a**^									0.0027																			1.000
Neonates	31	(	1.11	)	6,219	(	0.85	)		31	(	1.70	)	(	1.46	-	1.95	)	31	(	1.70	)	(	1.46	-	1.95	)	
Infants	7	(	0.25	)	3,700	(	0.51	)		6	(	0.33	)	(	0.22	-	0.44	)	6	(	0.33	)	(	0.22	-	0.44	)	
Children	22	(	0.79	)	9,908	(	1.35	)		16	(	0.88	)	(	0.70	-	1.06	)	16	(	0.88	)	(	0.70	-	1.06	)	
Adolescents	46	(	1.65	)	9,511	(	1.30	)		45	(	2.47	)	(	2.18	-	2.77	)	45	(	2.47	)	(	2.18	-	2.77	)	
Adults	1,125	(	40.24	)	393,197	(	53.67	)		1113	(	61.15	)	(	60.23	-	62.08	)	1113	(	61.15	)	(	60.23	-	62.08	)	
Elderly	614	(	21.96	)	234,965	(	32.07	)		609	(	33.46	)	(	32.57	-	34.35	)	609	(	33.46	)	(	32.57	-	34.35	)	
**Gender**									< .0001																			1.000
Female	1,120	(	40.06	)	407,029	(	55.56	)		907	(	49.84	)	(	48.89	-	50.78	)	907	(	49.84	)	(	48.89	-	50.78	)	
Male	1,351	(	48.32	)	311,051	(	42.46	)		913	(	50.16	)	(	49.22	-	51.11	)	913	(	50.16	)	(	49.22	-	51.11	)	
**Report source by person**									< .0001																			< .0001
Doctor	1,010	(	36.12	)	343,812	(	46.93	)		828	(	45.49	)	(	44.55	-	46.44	)	714	(	39.23	)	(	38.31	-	40.15	)	
Pharmacist	77	(	2.75	)	102,519	(	13.99	)		285	(	15.66	)	(	14.97	-	16.35	)	32	(	1.76	)	(	1.51	-	2.01	)	
Nurse	18	(	0.64	)	193,332	(	26.39	)		525	(	28.85	)	(	27.99	-	29.70	)	10	(	0.55	)	(	0.41	-	0.69	)	
Consumer	1,101	(	39.38	)	68,461	(	9.35	)		126	(	6.92	)	(	6.44	-	7.40	)	530	(	29.12	)	(	28.26	-	29.98	)	
Other	143	(	5.11	)	12,477	(	1.70	)		32	(	1.76	)	(	1.51	-	2.01	)	111	(	6.10	)	(	5.65	-	6.55	)	
Medical specialist^b^	14	(	0.50	)	398	(	0.05	)		0	(	0.00	)	(	0.00	-	0.00	)	14	(	0.77	)	(	0.60	-	0.93	)	
Missing	433	(	15.49	)	11,575	(	1.58	)		409	(	22.47	)	(	21.68	-	23.26	)	23	(	1.26	)	(	1.05	-	1.47	)	
**Report source by affiliation**									< .0001																			< .0001
RPVC^c^	23	(	0.82	)	351,280	(	47.95	)		985	(	54.12	)	(	53.18	-	55.06	)	23	(	1.26	)	(	1.05	-	1.47	)	
Pharmaceutical company	2,705	(	96.75	)	368,861	(	50.35	)		805	(	44.23	)	(	43.29	-	45.17	)	1735	(	95.33	)	(	94.93	-	95.73	)	
Medical institution	0	(	0.00	)	6,537	(	0.89	)		13	(	0.71	)	(	0.56	-	0.87	)	0	(	0.00	)	(	0.00	-	0.00	)	
Pharmacy	0	(	0.00	)	55	(	0.01	)		0	(	0.00	)	(	0.00	-	0.00	)	0	(	0.00	)	(	0.00	-	0.00	)	
Health center	0	(	0.00	)	4	(	0.00	)		0	(	0.00	)	(	0.00	-	0.00	)	0	(	0.00	)	(	0.00	-	0.00	)	
Consumer	68	(	2.43	)	5,668	(	0.77	)		17	(	0.93	)	(	0.75	-	1.12	)	62	(	3.41	)	(	3.06	-	3.75	)	
Other	0	(	0.00	)	169	(	0.02	)		0	(	0.00	)	(	0.00	-	0.00	)	0	(	0.00	)	(	0.00	-	0.00	)	
**Serious adverse event**									0.1377																			0.229
Yes	567	(	20.28	)	157,013	(	21.43	)		345	(	18.96	)	(	18.21	-	19.70	)	317	(	17.42	)	(	16.70	-	18.13	)	
No	2,229	(	79.72	)	575,561	(	78.57	)		1475	(	81.04	)	(	80.30	-	81.79	)	1503	(	82.58	)	(	81.87	-	83.30	)	

^a^:Neonates (<28 days); Infants (28 days–24 months); Children (24 months–12 years); Adolescents (12–19 years); Adults (19–65 years); Elderly (≥65 years)

^b^:Who practices one branch of medicine

^c.^Abbreviation: RPVC, regional pharmacovigilance center; TI, therapeutic ineffectiveness

^d.^95% CI of proportions are only shown for after age- and gender matched data

Prior to age group and gender matching, all covariates except adolescents and nurses in the logistic regression model were associated with therapeutic ineffectiveness, whereas after matching, those excluding pharmacists and nurses in the model were associated (Table 2). Pertaining to report source by person, others (adjusted ROR 6.09; 95% CI, 3.27 to 11.37) and consumers (adjusted ROR 3.98; 95% CI, 2.92 to 5.41) had a higher therapeutic ineffectiveness ROR, setting doctors as the reference group. As for report source by affiliation, consumers (adjusted ROR 55.84; 95% CI, 17.93 to 173.98) and pharmaceutical companies (adjusted ROR 60.10; 95% CI, 26.41 to 136.74) were more likely to report therapeutic ineffectiveness than the RPVC.

**Table 2:** Logistic regression model for the factors associated with therapeutic ineffectiveness occurrence rate according to the age group, gender, reporter, source of report, and serious adverse event from the KAERS database in 2016.

	Before age group, gender matching											After age group, gender matching^e^										
	TI Reports				ROR							TI Reports				ROR						
	N = 2,797 (%)				Crude	Adjusted (95% CIs)^d^						N = 1,820 (%)				Crude	Adjusted (95% CIs)^d^					
**Age group^a^**																						
Infants	31	(	1.11	)	REF	REF						31	(	1.70	)	REF	Exactly Same for Each Variable					
Neonates	7	(	0.25	)	0.38	0.30	(	0.12	-	0.72	)	6	(	0.33	)	1.00						
Children	22	(	0.79	)	0.45	0.45	(	0.24	-	0.83	)	16	(	0.88	)	1.00						
Adolescents	46	(	1.65	)	0.97	1.01	(	0.60	-	1.70	)	45	(	2.47	)	1.00						
Adults	1,125	(	40.24	)	0.57	0.42	(	0.29	-	0.61	)	1113	(	61.15	)	1.00						
Elderly	614	(	21.96	)	0.52	0.50	(	0.34	-	0.72	)	609	(	33.46	)	1.00						
**Gender**																						
Male	1,120	(	40.06	)	REF	REF						907	(	49.84	)	REF						
Female	1,351	(	48.32	)	0.63	0.72	(	0.64	-	0.80	)	913	(	50.16	)	1.00						
**Report source by person**																						
Doctor	1,010	(	36.12	)	REF	REF						828	(	45.49	)	REF	REF					
Pharmacist	77	(	2.75	)	0.26	4.88	(	3.21	-	7.42	)	285	(	15.66	)	0.12	1.54	(	0.63	-	3.76	)
Nurse	18	(	0.64	)	0.03	1.14	(	0.56	-	2.29	)	525	(	28.85	)	0.02	0.79	(	0.31	-	2.01	)
Consumer	1,101	(	39.38	)	5.47	4.75	(	4.21	-	5.35	)	126	(	6.92	)	4.43	3.98	(	2.92	-	5.41	)
Other	143	(	5.11	)	3.90	9.44	(	7.66	-	11.63	)	32	(	1.76	)	3.54	6.09	(	3.27	-	11.37	)
Medical specialist^b^	14	(	0.50	)	11.97	66.84	(	37.44	-	119.33	)	0	(	0.00	)	NA	NA	(	NA	-	NA	)
**Report source by affiliation**																						
RPVC^c^	23	(	0.82	)	REF	REF						985	(	54.12	)	REF	REF					
Pharmaceutical company	2,705	(	96.75	)	112.00	117.37	(	69.45	-	198.36	)	805	(	44.23	)	87.16	60.10	(	26.41	-	136.74	)
Consumer	68	(	2.43	)	183.23	85.35	(	47.41	-	153.63	)	17	(	0.93	)	161.73	55.84	(	17.93	-	173.98	)
**Serious adverse event**																						
Yes	567	(	20.28	)	REF	REF						345	(	18.96	)	REF	REF					
No	2,229	(	79.72	)	1.07	2.18	(	1.92	-	2.48	)	1,475	(	81.04	)	0.90	1.43	(	1.10	-	1.88	)

^a^:Neonates (<28 days); Infants (28 days–24 months); Children (24 months–12 years); Adolescents (12–19 years); Adults (19–65 years); Elderly (≥65 years)

^b^:Who practices one branch of medicine

^c^:Abbreviation: CI, confidence interval; ROR, reporting odds ratio; RPVC, regional pharmacovigilance center; TI, therapeutic ineffectiveness

^d.^95% CI are shown only for adjusted ROR

^e^Final Model (3,203 observations) adjusted by report source by person, report source by affiliation and serious adverse event (matched by age and gender)

Table 3 shows the three most frequently reported drugs and classes by age groups. Among all age groups, adults (61.15%) reported the highest number of reports. By age group, in the elderly, teriparatide, which is indicated for the treatment of osteoporosis, was the most reported drug with 192 reports. In adults, ciclopirox, which is an antifungal drug, had 126 reports and escitalopram, a selective serotonin reuptake inhibitors (SSRIs), had 52 reports. In adolescents, reports of methylprednisolone had four reports, and in adolescents and children, amphotericin B, paroxetine and bisacodyl, paracetamol had three and two reports, respectively. In both infants and neonates, pneumococcus antigen had three reports.

**Table 3:** Frequency of therapeutic ineffectiveness reports received by the KAERS in 2016 and the three most frequently reported generic name according to the matched age group.

Age group	N (%)				Generic Name (n^a^)	Therapeutic Indication	Induction Period^b^ (days; Mean ± SD)		
Neonates	31	(	1.70	)	Hemophilus influenzae B (4)	Haemophilus Influenzae Type B	947	±	455
					Ampicillin (3)	Bacterial Infection	378	±	321
					Chlorphenamine (3)	Hay Fever, Allergic Condition	238	±	203
Infants	6	(	0.33	)	Pneumococcus antigen (3)	Pneumococcal Disease	1	±	NA
					Amikacin (1)	Bacterial Infection	7	±	NA
					Levetiracetam (1)	Seizure Disorder	NA	±	NA
Children	16	(	0.88	)	Acetylcysteine (2)	Mucolytic/ Lung Surfactant	5	±	NA
					Bisacodyl (2)	Constipation, Bowel Cleanser	1	±	1
					Paracetamol (2)	Pain Relief	781	±	445
Adolescents	45	(	2.47	)	Methylprednisolone (4)	Corticosteroid- responsive Disorder	NA	±	NA
					Amphotericin B (3)	Fungal Infection	NA	±	NA
					Paroxetine (3)	OCD	NA	±	NA
Adults	1113	(	61.15	)	Ciclopirox (126)	Skin Infection	152	±	128
					Escitalopram (52)	Anxiety, OCD Mood Disorder	736	±	876
					Teriparatide (38)	Osteoporosis	237	±	162
Elderly	609	(	33.46	)	Teriparatide (192)	Osteoporosis	162	±	147
					Acetylsalicylic acid (25)	Pain Relief	1162	±	1233
					Indacaterol (23)	Asthma	397	±	301

^a.^ Number of therapeutic ineffectiveness reports

^b^. Induction period: time between the start of treatment and the report of ineffectiveness in days

Abbreviation: OCD, obsessive compulsory disorder; SD, standard deviation

Table 4 describes the frequently reported drugs according to gender. During 2016, the number of reports of therapeutic ineffectiveness by males (49.84%) and females (50.16%) was almost the same. Ciclopirox (49), teriparatide (40), escitalopram (30), and acetylsalicylic acid (24) were frequently reported medications by males. In contrast, teriparatide (179), ciclopirox (86), escitalopram (27), and fentanyl (27) were frequently noted in female reports.

**Table 4:** Frequency of therapeutic ineffectiveness reports reported in the KAERS in 2016 and the 10 most frequently reported generic name according to gender.

Gender	N (%)				Generic Name (n^a^)	Therapeutic Indication	Induction Period^b^ (days; Mean ± SD)		
Male	907	(	49.84	)	Ciclopirox (49)	Skin Infections	142	±	110
					Teriparatide (40)	Osteoporosis	94	±	88
					Escitalopram (30)	OCD, Anxiety, Mood Disorder	114	±	NA
					Acetylsalicylic acid (24)	Pain Relief	1268	±	1114
					Indacaterol (23)	Asthma	430	±	304
					Tramadol (22)	Pain Relief	14	±	37
					Fentanyl (22)	Pain Relief	18	±	60
					Sildenafil (20)	Erectile Dysfunction	877	±	1234
					Zolpidem (19)	Insomnia	1959	±	739
					Paroxetine (18)	OCD	1841	±	NA
Female	913	(	50.16	)	Teriparatide (179)	Osteoporosis	196	±	158
					Ciclopirox (86)	Skin Infections	127	±	141
					Escitalopram (27)	OCD, Anxiety, Mood Disorder	1353	±	NA
					Fentanyl (27)	Pain Relief	2	±	5
					Paroxetine (24)	OCD	1890	±	NA
					Tramadol & Paracetamol (23)	Pain Relief	18	±	28
					Propulsives (20)	Gastro-intestinal motility	479	±	579
					Alprazolam (17)	Anxiety Disorders	1564	±	510
					Tramadol (17)	Pain Relief	29	±	124
					Iohexol (14)	Imaging Agents	119	±	360

^a.^ Number of therapeutic ineffectiveness reports

^b^. Induction period: time between the start of treatment and the report of ineffectiveness in days

Abbreviation: OCD, obsessive compulsory disorder; SD, standard deviation

As for other ADRs reported in addition to therapeutic ineffectiveness, under WHO-ART’s system organ class (SOC) classification of gastro-intestinal system disorders, nausea was reported the most frequently reported ADR with therapeutic ineffectiveness (43.9%), followed by abdominal pain (19.7%) and vomiting (15.5%) (Table 5). Another notable frequency ADR reported together with therapeutic ineffectiveness was dizziness under the SOC class central and peripheral nervous system disorders, with 50 reports, which comprised 38.8% of ADRs within that SOC class. Among ADRs under the SOC class, cardiovascular disorders (general), there were surprisingly 14 reports of cardiac failure, a very severe ADR.

**Table 5:** Frequency of other ADRs reported together with therapeutic ineffectiveness reports reported in the KAERS.

WHO-ART^a^ Classification		N (%)	
System Organ Class	Preferred Term		
Gastro-intestinal system disorders	Nausea	116	43.9
	Abdominal Pain	52	19.7
	Vomiting	41	15.5
	Diarrhea	27	10.2
	Dyspepsia	16	6.1
	Colitis Ulcerative Aggravated	12	4.5
Secondary terms (events)	Medication Error	98	72.6
	Fall	13	9.6
	Inappropriate Schedule Of Drug Administration	13	9.6
	Incorrect Technique In Drug Usage Process	11	8.1
Body as a whole (general disorders)	Pain	36	26.9
	Abscess	20	14.9
	Leg Pain	19	14.2
	Asthenia	18	13.4
	Condition Aggravated	17	12.7
	Resistance	14	10.4
	Fever	10	7.5
Central and peripheral nervous system disorders	Dizziness	50	38.8
	Headache Vascular	37	28.7
	Meningitis	18	14.0
	Paresthesia	13	10.1
	Gait Abnormal	11	8.5
Application site disorders	Injection Site Bleeding	31	30.1
	Injection Site Pain	21	20.4
	Injection Site Bruising	14	13.6
	Injection Site Rash	14	13.6
	Application Site Reaction	13	12.6
	Injection Site Pruritus	10	9.7
Skin and appendages disorders	Alopecia	19	20.9
	Nail Discoloration	18	19.8
	Rash	16	17.6
	Nail Disorder	13	14.3
	Skin Exfoliation	13	14.3
	Pruritus	12	13.2
Psychiatric disorders	Suicide Attempt	19	25.3
	Anorexia	18	24.0
	Insomnia	16	21.3
	Depression	11	14.7
	Somnolence	11	14.7
Musculo-skeletal system disorders	Fracture	27	54.0
	Myalgia	13	26.0
	Skeletal Pain	10	20.0
Respiratory system disorders	Coughing	17	41.5
	Hypoxia	14	34.1
	Pneumonia	10	24.4
Cardiovascular disorders (general)	Cardiac Failure	14	50.0
	Hypertension Neonatal	14	50.0
Resistance mechanism disorders	Back Pain	20	100.0
Heart rate and rhythm disorders	Fibrillation Atrial	19	100.0
Hearing and vestibular disorders	Hearing Decreased	18	100.0
White cell and RES^a^ disorders	Granulocytopenia	18	100.0
Red blood cell disorders	Anemia	15	100.0
Urinary system disorders	Face Edema	13	100.0

^a^ Abbreviation: RES, Reticuloendothelial system; WHO-ART, World Health Organization Adverse Reactions Terminology

**Fig 3:**
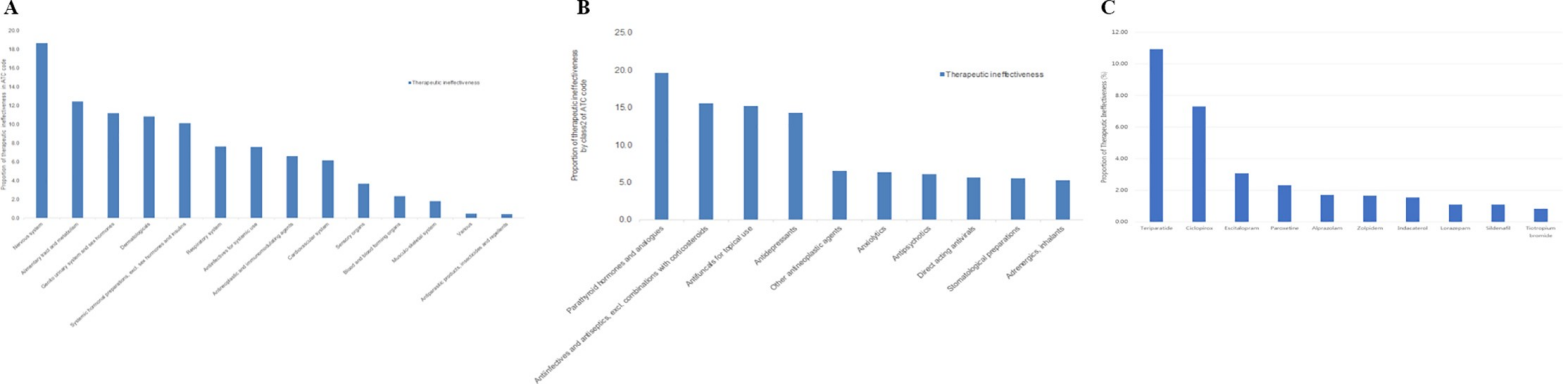
**Proportion of therapeutic ineffectiveness according to the (A) anatomical group of ATC code of WHO-ART, (B) pharmacological subgroups of ATC code of WHO-ART, (C) 10 most frequently reported generic names.** The figure is sorted in descending order of the proportion of the therapeutic ineffectiveness cases.

Fig 3A describes the frequent medication by anatomical groups. Listed in order are the nervous system (18.7%), alimentary tract and metabolism (12.5%), genitourinary system and gender hormones (11.2%), and dermatologicals (10.8%). Fig 3B illustrates the distribution according to the pharmacological subgroups. Parathyroid hormones and analogues (19.6%) were the most reported. Fig 3C shows the 10 most frequently reported drugs. From total reports recorded as therapeutic ineffectiveness, a teriparatide accounted for 11.0%, followed by ciclopirox 7.3% and escitalopram 3.1%.

Our study revealed the reporting trends in therapeutic ineffectiveness by year and its characteristics. For the 17-year period (2000–2016), the proportion of therapeutic ineffectiveness ranged from 0.0% to 3.7%, where for the recent four years, it ranged from 1.0 to 1.3% from 2013 to 2016. The categories of the Asia-Pacific Economic Cooperation (APEC) survey tool contain therapeutic ineffectiveness, which has been utilized in a previous study [8]. Among 21 countries surveyed, seven countries (Philippine, USA, Brunei, Peru, Indonesia, Chile, Malaysia) responded to the therapeutic ineffectiveness and medication errors. The Philippines had approximately 16% of therapeutic ineffectiveness reports, the USA had 5%, and the others less than 5% [9]. Reports involving therapeutic ineffectiveness changed continuously from 2000 to 2016. From a total of 228,939 reports, 2,797 (1.2%) were reported as therapeutic ineffectiveness in the KAERS database in 2016. Unlike Korea, the most frequently reported AE from the US Food and Drug Administration’s AE Reporting System (FAERS) was found to be drug ineffective, with 6.4% [10]. The differences in such findings between the two nations may attributed to the source of reports as consumers comprise the highest proportion of reporters in the US; whereas in Korea, the role of healthcare professionals and RPVCs are high. Therapeutic ineffectiveness-inducing drugs were concentrated in silent diseases, such as psychiatric and osteoporosis drugs.

There are conceptual differences present between AE and therapeutic ineffectiveness. AEs mean any untoward medical occurrences that may be present during treatment with a pharmaceutical product, which does not necessarily have a causal relationship with the treatment. On the other hand, therapeutic ineffectiveness means that there were no pharmaceutical reactions in the person who took the medicine [2]; therapeutic ineffectiveness is a subset of AE. Therapeutic ineffectiveness have been reported continuously; in the most recently published paper, over 5,454 types of unique AEs were reported to be associated with painkillers, and more frequently encountered AE included drug ineffectiveness (7.81%) [11]. There may be a variety of reasons to why therapeutic ineffectiveness may occur where some potential reasons may be: irrational use, drug’s pharmacokinetics characteristics or patient variability, drug interactions, tolerance, resistance, tachyphylaxis, or pharmaceutical defects such as adulterated, substandard or counterfeit drugs, which could in turn lead to the report of therapeutic ineffectiveness [1, 12]. As multiple reasons contribute to the reporting of therapeutic ineffectiveness, the growth of its respective report’s proportion is inevitable but rather, explainable and understandable, as seen from our results.

Our study showed that reporting therapeutic ineffectiveness by consumers had higher proportion than that of doctors (adjusted ROR 3.98; 95% CI, 2.92 to 5.41). Indeed, there is controversy regarding whether patients and professional reports, which are related to drugs, are of similar quality or not in the context of professional doubt regarding the quality of patient reports of side-effects [13–15]. Although the exact underlying reason is not certain, many interpretations may be possible. First, when drugs are required for long time periods in order for effects to appear, patients may feel that drugs they have taken had no effect. Such situations may make patients feel the medication is not therapeutically effective unlike the opinions of healthcare professionals. In cases that are unable to cure or alleviate symptoms for the consumer, because of their own problem, they respond with much more sensitivity and more quickly. As seen in Tables 3 and 4, the induction period in days are shown for the top three and 10 drugs reported with therapeutic ineffectiveness. For teriparatide, an osteoporosis drug, the mean induction period, the time taken from treatment commencement to report of therapeutic ineffectiveness) was very different for males and females with 94 and 196 days, respectively, whereas for ciclopirox, a drug used for skin infections, the induction period was rather in close proximity with 142 and 127 days for males and females, respectively. Second, adherence issues for the medication may affect our current results. Adherence rates are typically higher among patients with acute conditions, as compared with adherence rates with chronic conditions [16–18]. On close inspection, related to consumer’s adherence to the medications, medical adherence of the patients could affect the pharmacological effects [19], and medical adherence of anti-osteoporosis drug users was poor [20]. For these reasons, teriparatide and ciclopirox were reported the most for therapeutic ineffectiveness among drugs. A third factor is using drugs manufactured with poor quality. Use of counterfeit (poor-quality drugs) drugs, and the consequent failure of patients to improve, have led to false reports of drug resistance to disease [21]. Using poor-quality drugs is associated with local regulations, income and literacy rates [22].

Our study demonstrated that the relative frequency of therapeutic ineffectiveness events in neonates is rather high. In addition, a research conducted in South Korea showed that the risk of medication errors in children was 2.73 times (95% CI, 2.35 to 3.17) higher than that of adult medication errors [4]. Therapeutic ineffectiveness and high reporting rate of medication errors in pediatric medicine may be explained by the unclear guidelines in pediatrics. Due to the fact that there are no precise guidelines for pediatrics, the incident frequently occurs for a dose higher or lower than that actually required is administered to a child. In this respect, there is a possibility that therapeutic ineffectiveness or ADRs may occur in pediatrics more frequently.

Although there was no gender difference in the proportion of who reported therapeutic ineffectiveness, frequently reported drugs regarding therapeutic ineffectiveness differed. From our results, ciclopirox and teriparatide were reported the most, likely owing to the patient’s age. As for escitalopram, despite this very drug considered to be one of the most effective drug within its class, it was reported as the third most reported drug for both genders [23]. We believe that this may be due to the excessively high consumption of escitalopram regardless of gender in Korea. With respect to gender, both showed similar proportion of therapeutic ineffectiveness in analgesics and benzodiazepines. We believe that this is due to tolerance; these drugs are used continually and need higher doses to reach the same desired effects over time [24].

There are several strengths in this study. First, to our knowledge, our study is the first paper on therapeutic ineffectiveness trends. Therefore, our study could be utilized as a fundamental study for future research on therapeutic ineffectiveness. Secondly, we show the results of the trends in therapeutic ineffectiveness events with confidence owing to the utilization of a large and representative of the KAERS database. In addition, groups of patients reporting the most therapeutic ineffectiveness events, demographic characteristics, such as age and gender, were also considered and accounted for. Regarding spontaneous reporting systems, every country is different as drugs frequently used vary from country to country and biological differences exist from one ethnic group to another. Finally, we speculate that there are various factors that may affect therapeutic ineffectiveness, such as irrational use, drug´s pharmacokinetics characteristics and patient variability, which may, under special circumstance, induce the report of ineffectiveness.

Despite our strengths, results of our study should be interpreted with caution as the following limitations are present. First, therapeutic ineffectiveness events may be subjective as the most commonly reported medications for therapeutic ineffectiveness are osteoporosis and psychiatric drugs. Second, general disadvantages are present with the use of spontaneous reporting data. Only a small percentage of actual therapeutic ineffectiveness reports have been reported, as the reporting rate is not perfect. Moreover, several limitations are present in spontaneous reporting data, which contains limited clinical data and not all ADRs are reported to the KAERS database.

## Conclusions

Among therapeutic ineffectiveness reported to the KAERS database, the proportion of therapeutic ineffectiveness ranged from 1.0 to 1.3% from 2013 to 2016. We found that consumers were approximately 4-fold more likely to report therapeutic ineffectiveness compared with doctors, and the most frequently reported drugs as having therapeutic ineffectiveness were teriparatide, ciclopirox, and escitalopram. With regards to issues concerning drug safety, AE reports on therapeutic ineffectiveness may be used as scientific evidence and tool for either reevaluation or review of the respective drug by conduction of further clinical trials in order to confirm of its therapeutic effects. To date, despite minimal to even null interest in therapeutic ineffectiveness worldwide, results of this study provides an opportunity for researchers to gain interest in developing future studies, in turn acting as a catalyst for accumulation of findings on top of ours.
